# 1425. Changes in Clinical Presentation of TB due to the COVID-19 pandemic

**DOI:** 10.1093/ofid/ofac492.1254

**Published:** 2022-12-15

**Authors:** Nazia Khan, Surksha Sirichand, Xianhong Xie, Jacqueline M Achkar

**Affiliations:** Montefiore Medical Center, Bronx, New York; Montefiore Medical Center, Bronx, New York; Albert Einstein College of Medicine, Bronx, New York; Albert Einstein College of Medicine, Bronx, New York

## Abstract

**Background:**

In 2020/21, deaths due to active tuberculosis (TB) increased globally for the first time in decades with concomitant global decline in TB detection rates, suggesting that delay in TB diagnosis during the COVID-19 pandemic is associated with increased mortality. In the US and New York City, 20% decline in TB cases was reported in 2020/21. We aimed to compare symptom duration, sputum microscopy and radiographic findings in patients with newly diagnosed TB at Montefiore Medical Center (MMC) in the Bronx, New York, before and during the COVID-19 pandemic. We hypothesized that patients during the COVID-19 pandemic present with signs of more advanced TB than before.

**Methods:**

Using a cross-sectional study design, we retrospectively reviewed medical records of TB patients identified through microbiology lab records from 11/1/2018 to 3/11/2022 and stratified by admission before (11/1/2018–2/29/2020) and during (3/1/2020–3/11/2022) the COVID-19 pandemic. Inclusion criteria were age ≥18 years, admission to an MMC hospital, and new diagnosis of culture-confirmed TB.

**Results:**

We identified 24 TB patients who presented before and 24 during the pandemic. About 1.7 new TB cases were diagnosed monthly before vs 1.0 during the pandemic, an >40% decline. Patients had both pulmonary and/or extrapulmonary manifestations without differences between groups. There were no significant differences in demographics and comorbidities between the two groups aside from diabetes, which was higher in the pre-COVID group (p = 0.03). Two TB patients had a prior history of COVID and one developed nosocomial COVID during the admission. There was no difference in mortality between groups. Patients with pulmonary manifestations had higher sputum AFB smear positivity (p=0.14) and significantly higher occurrence of multilobar or miliary infiltrates on chest X-ray during compared to before COVID (p = 0.01; Table 1).

**Table 1.**
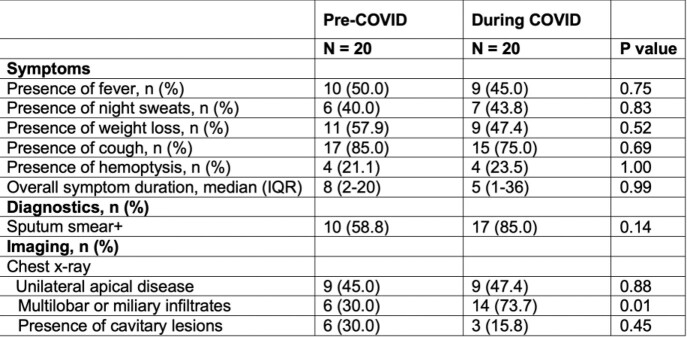
Symptoms and diagnostics on initial presentation of patients with pulmonary TB before and during the COVID-19 pandemic

Depending on distribution, t-tests or Mann Whitney U tests were used for continuous and Chi-square tests or Fisher’s exact tests for categorical variables. Sputum AFB smears results are reported for the initial 3 smears.

**Conclusion:**

Our findings show >40% decline in patients presenting with TB in the Bronx during vs before the COVID-19 pandemic and suggests patients presented with more advanced disease than before the pandemic. Whether COVID-19 could have contributed to this remains to be investigated. Our results have implications for public health and emphasizes the need for earlier identification of TB.

**Disclosures:**

**All Authors**: No reported disclosures.

